# Shifting our stance for current COVID-19 outbreaks: A global response to an international pandemic

**DOI:** 10.7189/jogh.11.03123

**Published:** 2021-12-11

**Authors:** Jed Keenan Obra, Bryant Lin, Latha Palaniappan, Gloria S Kim

**Affiliations:** 1 Stanford Center for Asian Health Research and Education, Stanford University School of Medicine, Stanford, California, USA; 2 Department of Medicine, Division of Primary Care and Population Health, Stanford University School of Medicine, Stanford, California, USA; 3 Department of Medicine, Division of Cardiovascular Medicine, Stanford University School of Medicine, Stanford, California, USA

The current COVID pandemic, and the quick spread of infectious diseases in general, remind us how interconnected we are as human beings on our planet. The United States (US) and other developed countries with strong vaccination rates should not consider themselves immune from the current pandemic until more people in the world are protected. With the Delta variant threatening increased transmission and reduced effectiveness of current vaccines [1], vaccine administration alone is not the total solution to achieve herd immunity within developed countries either. Especially in pockets of the world where public health is not coordinated and vulnerable populations face difficulties accessing health care and vaccines, the pandemic has avoided the only weapon developed against it and continues to ricochet around the world. Unless world leaders see the world community as their own and join to fight the contagion as part of a united global front, COVID-19's destructive effects will continue to wash over and ripple everywhere, including the US. The next pandemic is happening now, and it is time for the US leaders to join nationally and internationally in a war not against each other, but against a common enemy: the global pandemic.

In May 2021, Stanford University School of Medicine convened over 40 global researchers, physicians, and health advocates and 500 health attendees across 12 time zones to share lessons learned from their country’s respective public health efforts during the first year of the COVID-19 pandemic.

## VACCINE HESITANCY

One of the main points our speakers emphasized was the importance of trust for public health success [2]. Within the US, vaccine hesitancy has lowered demand for the vaccine and created a large surplus among vaccination sites [3]. Much of this hesitancy seems to arise from community distrust in government and health organizations due to media misinformation and conflicting media perspectives, as well as safety and efficacy concerns from the novel approaches that investigators used to create the vaccine [4]. Furthermore, the issue of vaccination has become very politicized. The ongoing conflict within the US two-party political system with polarized views regarding the power of the state over individual rights extends into public health, with some right-wing members rejecting mandatory masks and calls for vaccination as a subversive plot [5]. “Fake news” and misguided false attacks on scientists were allowed to flourish on social media, leading to confusion all around. In addition, there were some scientists who were willing to ignore data and promote views that were favored by some political leaders [6]. Some of the highest rates of vaccination within the USA are seen in areas where there is political alignment and community support of health authorities [7], and this approach should be heavily considered in the goal for herd immunity.

## EQUITABLE VACCINE ACCESSIBILITY

While hesitancy is being rightfully discussed among public health leaders, accessibility must be equally considered for poor countries and poor local communities who lack resources to compete against their wealthier counterparts. These disadvantaged may be willing and wish to be vaccinated, but have limited access, either due to inadequate supply, transportation or time to avail themselves. They remain the most vulnerable to new outbreaks. Outbreaks of variants have been shown to spread widely and quickly throughout cities, counties, states, countries. It is a global pandemic. While wealthy countries bid for most vaccines created by manufacturers [8], low-income and unfortunately the most vulnerable countries are left to vaccinate only a small proportion of its citizens. With the biggest threat of the pandemic lying within new outbreaks among the most vulnerable, accountability for national leaders should no longer be restricted to within their own community or even their own country. The local community is intertwined within the global community. What happens locally can be repeated globally, many times over. We can learn best practices from local areas that have fared well in public health and transmit this knowledge worldwide. At the same time, what is learned globally, can be shared and applied locally.

## MANDATE FOR ACTION

The inability to approach herd immunity at a pace faster than the spread of COVID-19 variants calls for an adjustment in the roles of leadership during a health crisis. As it is becoming increasingly clear that the pandemic is an international problem that requires a global solution, the necessity to acknowledge and empower public health leaders to think and act from a shared problem is clear. This requires that knowledge is shared among local, national, and international communities. Modern technology allows for this to be done very quickly [2]. Currently, knowledge sharing is conducted as a matter of goodwill. To give more strength to the mandate for the world to share responsibility, there may need to be an official pact and pledge signed by members of the World Health Organization (WHO) to work collaboratively on matters of international and global concern. Such collaboration has proven to be a very powerful tool as seen through the World Health Assembly, a forum for countries to share information and make collective decisions [9]. The warp speed at which the COVID-19 vaccines were developed would not have been possible without the Chinese scientists sharing the genome information as quickly and completely as they did. Support for the WHO should come from all countries for the sake of their own national health. Knowledge sharing on a global scale will allow national leaders to combine local and international perspectives and build the most informed perspective prior to building and implementing their public health agendas and recommendations [9]. With international organizations such as the WHO aggregating and disseminating information and recommendations to countries, national leaders can appropriately act on these recommendations, appropriate to the local culture of their countries and communities.

## RELIABLE COMMUNICATION OF DATA

Just as there is a greater need for communication between health and government officials on a national and international level, there is also a need for fast and reliable public communication during crises. This would include sharing the results of public surveillance and community disease trends, the mainstay of public health. Knowledge sharing reduces the risk of presenting fragmented and contradictory information of health risks towards vulnerable and at-risk populations [10]. From our conference, we found that integrating past data and current real time data are crucial for identifying community risk and establishing public policy priorities, but it also requires translating into a digestible manner for community audiences to apply it to their lives [2]. The onus for this lies within national agencies who believe in science to be cautious of misinterpretations and maintain strong relationships with local leaders who will be educating their communities.

**Figure:**
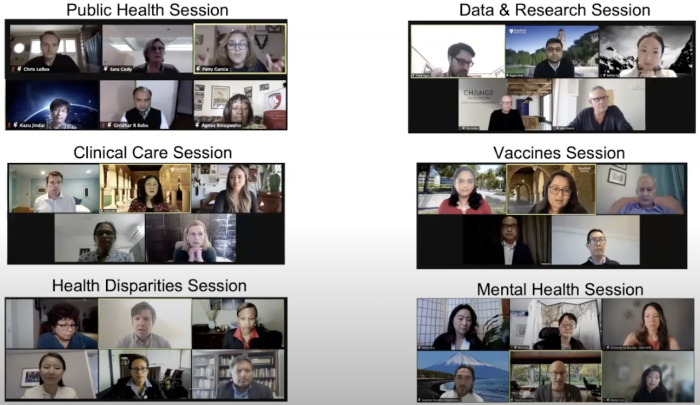
Photo: Panels of international speakers from multiple conference sessions at Stanford University's 2021 International COVID-19 Conference (from the team's collection, used with permission).

## REACHING HERD IMMUNITY

Herd immunity has become increasingly challenging with the spread of new COVID-19 variants, but it is not impossible. Vaccinating our children is key. Herd immunity to measles and the elimination of smallpox was a victory from high efficacy vaccines, but it took over 40 years for measles and smallpox to reach this [11,12] and is strongly attributed to the incorporation of vaccines into the schedule for routine childhood vaccination [13]. With current vaccine administration rates, we are reaching herd immunity with COVID-19 much faster than measles and smallpox but are still far from achieving it. Vaccinated individuals should continue social distancing protocols and mask-wearing rules to protect remaining individuals yet to receive the vaccine. The US must especially focus on local communities and states with lower vaccination rates. Preventative measures should be complemented with proactive measures such as empowering local health systems of poorer countries for which the vaccine is still inaccessible and COVID-19 variants have a higher chance of mutating. With proper surveillance and a trained group of health workers to handle newly identified variants immediately, COVID-19 variants may have a much smaller risk of spreading to nearby regions, communities, and countries [14]. While all these actions necessitate collaboration on the local and international level, the US can bring these goals within striking distance if partisan politics unite in acknowledging these as a priority.

## BUILDING TRUST

As global leaders work together to be on the same page in caring for the global community as well as their own, garnering and maintaining public support of public health agendas will be essential to success in tackling the current pandemic and future pandemic crises [2]. Rwanda and New Zealand’s successful public health campaigns drew from large public support of government restrictions, and it is important to note that these countries highly differ in both income and population [15,16]. While Rwanda’s shortage of vaccines has rendered the country unable to vaccinate more than 5% of its population (17.9% for New Zealand and 52.4% for the US), COVID-19 case rates have remained stable with public health compliance as of August 2021 [17,18]. In the US where perspectives in receiving the vaccine are divisive, public health agendas struggle to approach the 90% benchmark estimated by public health experts needed to achieve herd immunity [19]. This support boils down to community trust and can be garnered from establishing well-trusted national organizations that believe in science and are supported by both major US political parties, which will encourage US citizens to comply with public health recommendations. These organizations must empower local leaders who understand how to make the vaccine accessible for their communities and are able to address individual concerns regarding vaccine treatment. Trust is built over years and while it must start now in our ongoing pandemic, it must carry to the next crises. The US will find success in its agendas if it can earn the trust of most of its citizens, and this will not only position the US to help set global standards, but it will also create a blueprint for all future crises to be tackled on a global front.

Building trust within communities and countries also means addressing the roots of distrust among community members. Trauma brought by structural racism and inequity decreases community trust in individuals, institutions, and information. COVID-19 has spotlighted these socioeconomic and racial/ethnic disparities through differences in health outcomes [2]. In these communities, it is understandable that they would hesitate to follow leaders who have dismissed their presence and needs. As humans on the global scale, we differ in regional cultures and social norms, but it is through these differences that we can find bright spots among local communities that can be shared on the global scale. Native American tribes, a community among the hardest hit by COVID-19, celebrated a pandemic success story which tribal leaders attribute to tribal sovereignty where they were given flexibility for how they distribute the vaccine [7]. In communities where there is distrust in the health care system, the task of implementing public health recommendations must be entrusted to trusted local leaders. Access does not mean acceptance, but both are necessary for us to beat the following waves of this pandemic. Newfound successes discovered by addressing disparity gaps equips the US with more knowledge for successfully providing greater access to information, education, and technology – invaluable assets to handling future pandemic threats – to the entire US population. Closing disparity gaps will be key to equitably uplifting all communities, and it is only by empowering everyone that we can all be strong.

## MOVING FORWARD

“If you want to go fast, go alone; if you want to go far, go together” *– African proverb.*

Fighting the pandemic not as a country but rather as part of a bigger community – a global one – is necessary to overcome this pandemic. COVID-19 outbreaks will continue to ping pong around the world until we uplift all countries including our own, and the key to our success lies in knowledge sharing, public health compliance, and community trust. The US especially must take a collectivist stance that focuses on altruism globally if we are to end the pandemic swiftly. Through transparent communication, local leaders, empowered by resources provided by national and international leaders, will be able to spearhead efforts to reach herd immunity and build trust among skeptical communities. Local inclusion of all communities within a nation and global inclusion of all countries in the international pandemic relief agenda is necessary to win this global war against COVID-19. Once countries empower themselves and each other, the pandemic will be stopped in its tracks and set precedents for a rapid, effective, local and global response for future crises. The future is now.
